# Risk Factors for HIV/Syphilis Infection and Male Circumcision Practices and Preferences among Men Who Have Sex with Men in China

**DOI:** 10.1155/2014/498987

**Published:** 2014-03-30

**Authors:** Yali Zeng, Linglin Zhang, Tian Li, Wenhong Lai, Yujiang Jia, Muktar H. Aliyu, Mai Do, Xiaodong Wang, Delin Han, Wanli Huang, Shuping Du, Jie Xu, Jiushun Zhou, Shu Liang, Fei Yu, Yanqing Zhang

**Affiliations:** ^1^Sichuan Provincial Center of Disease Control and Prevention, Chengdu 610041, China; ^2^Vanderbilt Institute for Global Health, Vanderbilt University, Nashville, TN 37027, USA; ^3^School of Public Health and Tropical Medicine, Tulane University, New Orleans, LA 70112, USA; ^4^Chengdu Gay Community Care Organization, Chengdu 610021, China; ^5^Chengdu Center of Disease Control and Prevention, Chengdu 610041, China; ^6^Sichuan Construction Workers Hospital, Chengdu 610081, China; ^7^National Center for AIDS/STD Control and Prevention, Chinese Center for Disease Control and Prevention, Beijing 102206, China; ^8^Medical School, Tulane University, New Orleans, LA 70112, USA

## Abstract

*Objective*. To investigate factors associated with HIV infection and the frequency and willingness of male circumcision among men who have sex with men (MSM) in Chengdu city, China. *Methods*. A cross-sectional survey provided information on participants' demographics, risk behaviors, circumcision, and uptake of HIV prevention services. *Results*. Of 570 participants, 13.3% were infected with HIV and 15.9% with syphilis. An estimated 43.0% of respondents reported having unprotected receptive anal intercourse, and 58.9% reported having ≥2 male sexual partners in the past 6 months. Multivariable logistic regression revealed that syphilis, more male sex partners, predominantly receptive anal intercourse, and exclusively receptive male sex were associated with HIV infection. Higher level of education and peer education service were inversely associated with HIV infection. Nearly a fifth (18.0%) of participants were circumcised. More than half of uncircumcised participants expressed willingness to be circumcised. *Conclusion*. This study reveals a high prevalence of HIV and syphilis among MSM in Chengdu province of China. The frequency of unprotected receptive anal intercourse and multiple male sexual partnerships highlight the urgency for an effective comprehensive HIV prevention strategy. Although the willingness to accept male circumcision (MC) is high, further research is needed to assess the protective effective of MC among MSM.

## 1. Introduction

Since reports of the HIV virus began to emerge in the United States in the 1980s, the HIV epidemic has frequently been linked to men who have sex with men (MSM) [1]. Sexual transmission among MSM accounts for the majority of prevalent AIDS cases in Western Europe, United States, Canada, Australia, and New Zealand. In Africa, Asia, and Latin America, the prevalence of HIV infection has increased rapidly in recent years [2]. Although HIV epidemics in low and middle income countries are mainly driven by heterosexual sex, injection drug use, and/or contaminated blood collection and transfusion, with MSM comprising a small share of all HIV cases, recent data shows rapid increases in the HIV epidemic among MSM in Asia, Africa, South America, Eastern Europe, and Central Asia [3]. In China, meta-analyses among MSM indicated HIV prevalence rates in the range of 3.2%–15.8% for studies conducted during 2005–2008 [4] and a pooled syphilis prevalence of 9.1% for studies conducted from 2001 to 2008 [5]. A large national survey of 47,231 MSM in China indicated HIV and syphilis prevalence rates of 4.9% and 11.8% in 2008, respectively [6]. Data consistently indicated unprotected male-to-male sexual contact has become one of the major transmission routes for HIV in China [7].

Chengdu is one of the large metropolitan cities in Southwest China. There are estimated 71,000 MSM among its 4.5 million residents in 2007, among whom at least 10,000 are active members of the homosexual social network [8]. HIV/AIDS surveillance indicates that HIV prevalence increased rapidly from 1.06% (2/189) in 2004 [9] to 11.2% (51/456) in 2008 [10], a rate that is twice as high as the national average HIV prevalence among MSM [6].

Antiretroviral treatment and treatment of sexually transmitted infections (STIs) have been approved as effective prevention approaches for HIV, and increasingly promoted as a means of reducing HIV transmission among MSM [11–15]. There is compelling evidence that male circumcision reduces the risk of heterosexually acquired HIV infection in men. Three randomized clinical trials demonstrated that male circumcision (MC) reduced HIV acquisition risk among heterosexual men by approximately 60% [16–18]. The World Health Organization (WHO) and UNAIDS recommend male circumcision as an additional, important strategy for the prevention of heterosexually acquired HIV infection in men. Several studies of MSM who predominantly practice insertive anal intercourse (IAI) suggest some protective effect of male circumcision, as reported in Peru [19] South Africa [20], and Australia [21]. However, most studies have not found an association between HIV prevalence, incidence, and circumcision, likely because of multiple factors, including evidence that circumcision will not reduce HIV acquisition through unprotected receptive anal intercourse (RAI). In addition, African data may not be readily generalizable to MSM [22] and the potential efficacy of circumcision in reducing HIV infection among MSM is still unclear [23, 24].

In China, MC is not commonly practiced; while the prevalence of MC worldwide is almost 30%, it is only 5% among Chinese males [25]. Data on circumcision among Chinese adult men is very limited. There is no data available on MSM male circumcision in Sichuan. The purpose of this study was to assess the prevalence of HIV and syphilis, investigate factors associated with HIV infection, and the frequency and willingness to undergo male circumcision among MSM in Chengdu city of China.

## 2. Methods

### 2.1. Enrollment of Participants

A cross-sectional survey was conducted in Chengdu from June to September 2009. MSM eligible for the study included men 18–69 years of age who reported engaging in sex with men in the previous year.

Stratified-snowball sampling was used to recruit participants from the Chengdu MSM community [6]. A formative assessment was conducted to determine the sizes of the MSM populations who most often seek sex partners at social venues including bars, tea houses, dance halls, public bathhouses/saunas, parks, public restrooms, and internet sites. Each venue represented a stratum of MSM to be surveyed. A community based organization, Chengdu Gay Community Organization (CGCO), helped to identify “seed” individuals (seed eligibility criteria: (1) meeting study criteria and (2) having a broad social network), who then began the referral chain. Our study recruited 18 seeds and each one was given an unlimited number of referral coupons to recruit other subgroup members. These individuals in turn were asked to provide information on other subgroup members. Recruitment ceased when the required sample size was reached [26]. All participants except for the seeds were required to present a coupon at their visit to be considered eligible for the survey. CGCO helped to identify, recruit, and record each participant to avoid recruiting the same participants from different seeds. Each participant who completed the survey received as incentive a US $8 coupon as compensation for transportation costs.

### 2.2. Interview and Measures

The study included 8 steps, namely, participant identification, obtaining informed consent, pretest counseling, blood collection, questionnaire administration, genital exam, HIV and Syphilis rapid testing, and posttest counseling. Interview settings had two private interview/counseling rooms, two testing rooms, and one waiting room, which was located in Sichuan construction workers' hospital (designated for Chengdu MSM STI testing and treatment services). All participants completed the written questionnaire via a face-to-face interview. The information collected in the questionnaire included demographic characteristics; HIV knowledge and attitudes; sexual history with other men and women, including unprotected anal intercourse, commercial sex; self-reported STD infection history, circumcision history, and history of receipt of HIV prevention services. A blood sample (approximately 8 mL) was collected from each individual and tested immediately on-site for HIV and current, active syphilis infection (not lifetime exposure). The rapid serological results were provided to the individual directly. CGCO's trained health counselors were responsible for pre- and posttest counseling. Counseling was conducted in an individual setting to assure client's privacy and confidentiality. During pretest counseling, consultants helped the participant to get mentally prepared through counseling on benefits of HIV testing and explaining the testing process. Pretest counseling lasted about 10 minutes. During the postcounseling, consultants provided test results, counseled on HIV prevention and HIV/STD risk reduction and provided appropriate referrals and take home information. Participants who tested positive were referred to the peer support project. Posttest counseling also gave participants sufficient time to react and to obtain emotional support, so it took about 15 minutes on average. Samples yielding inconclusive HIV results from rapid, on-site tests were subjected to confirmatory testing and participants were asked to return to receive their results. A genital examination was conducted as well.

### 2.3. Statistical Analysis

HIV prevalence was calculated by dividing the sum of all confirmed cases by all participants. In addition, the prevalence of circumcision, willingness to be circumcised, and the relationship between MC and HIV infection were analyzed. Univariate and multivariate logistic regressions analyses were used to assess the association between risk factors and HIV infection. Variables significant at a level of 0.2 in univariate logistic regression analyses were fitted into multivariate models. Multivariate logistic regression models were constructed to identify independent risk factors for HIV infection, while controlling for potential confounding factors. Missing values were treated as separate categories for clarity but otherwise received no special treatment. All regression models were run using complete cases. All statistical analyses were performed using SAS v9.2 for Windows (SAS Institute, Cary, NC).

### 2.4. Ethical Approval

This study was reviewed and approved by the Institutional Review Board of the National Center for AIDS/STD Control and Prevention, China Center for Disease Control and Prevention. Subjects provided signed informed consent and were assigned unique identification numbers so that anonymity could be maintained yet double testing prevented.

## 3. Results

### 3.1. Characteristics of Participants

A total of 570 eligible participants completed the survey. Two participants declined to participate in the study. The age of participants ranged from 18 to 69 years with median age of 26.5 years. Approximately 40% of participants were under 25 years of age. About 60% of participants had college or higher levels of education, and nearly three-quarters (71.4%) were never married. Approximately 46% were registered as Chengdu residents and nearly two-thirds (65.3%) self-identified as homosexual. Less than 30% (161/570) of respondents self-identified as bisexual, 5% did not state their sexual orientation or “did not know”, and 1% self-identified as heterosexual. The most common venues to find male sexual partners included the internet (59.1%), bars (18.2%), public bathhouses (10.5%), peer referral (6.7%), and parks or public restrooms (5.4%).

### 3.2. Sexual Behaviors

The age of sexual debut with a man ranged from 11 to 63 years with a median of 20 years. In terms of MSM anal sex, 34.2% of participants exclusively engaged in insertive anal intercourse (IAI), 25.3% predominantly (>50%) engaged in insertive anal intercourse, 20.7% exclusively engaged in receptive anal intercourse (RAI), and 19.4% predominantly engaged in receptive anal intercourse. In the 6 months preceding the study, the median number of male partners was 2 (range: 1 to 100), 16.5% of respondents reported having female sexual partners, and 21 (3.7%) admitted to paying for sex with “Money Boys” (MBs), while 26 respondents (4.6%) sold sex to male clients. The rate of consistent condom use (always used condoms when having sex) varied by type of sexual partners; 50.0% condom use while selling sex to male partners and 34.4% while having sex with a female partner. About two-thirds of respondents used a condom in the last anal sex whereas 223 participants (43.0%) admitted to engaging in unprotect RAI.

### 3.3. Prevalence of HIV and Syphilis and Uptake of Related Prevention Services

HIV prevalence in the study population was 13.3% and syphilis prevalence was 15.9%. Syphilis-positive MSM had the highest HIV prevalence (37.8%) (Table 1). In the previous year, 84.0% of participants reported ever receiving HIV related services, 58.1% received peer education, 56.5% received HIV/STI knowledge booklet, 50.5% received free condoms, 46.8% received HIV testing and counseling, 46.1% received free lubricants, and 27.7% received STI testing. One-fifth of participants reported ever having STI-related symptoms in the past year (Table 2).

### 3.4. Factors Associated with HIV Infection

Multivariable logistic regression analysis suggested that a positive syphilis result, (AOR = 6.9; 95%CI: 3.5–13.5), having more male sex partners (AOR = 6.2; 95%CI = 2.0–19.1), predominantly engaging in receptive anal intercourse (AOR = 2.9; 95%CI: 1.2–6.9), and exclusively engaging in receptive male sex (AOR = 3.9; 95%CI: 1.6–9.3), was independently associated with HIV infection. Having a college education (AOR = 0.4; 95%CI: 0.2–0.9) and receipt of peer education services in the last year (AOR = 0.5; 95%CI: 0.3–0.8) were protective of HIV infection (Table 3).

In the entire group of MSM, circumcision was not associated with HIV, but the prevalence of syphilis was significantly lower among men who were circumcised than those who were uncircumcised (7.6% versus 17.5% resp., *P* < 0.05). These findings stayed the same after stratifying by participants' sexual role (IAI or RAI) in the past six months (Table 4).

### 3.5. Circumcision

Nearly a fifth of respondents (18.2%) reported having been circumcised, which was confirmed by clinical examination. The median age of circumcised participants was 20 years and ranged from 3 to 44 years. Reasons provided by circumcised MSM for the circumcision included a redundant foreskin (58.7%); 13.2% prevention of HIV and STIs (13.2%); personal hygiene (8.8%); to enhance sexual pleasure (5.9%) and cosmetic reasons to improve one's physical appearance (4.4%). The clinical examination indicated that more than half (53.2%) of individuals had foreskin problems, such as phimosis (1.1%), redundant foreskin (28.8%), and paraphimosis (11.6%). Among 504 uncircumcised men, 56.9% reported that they were willing to be circumcised; 30.4% were absolutely not willing to undergo circumcision, and 12.7% were not sure. For those participants who would accept male circumcision, the main reasons included prevention of HIV and STDs (49.0%), redundant foreskin (48.3%), enhance sexual pleasure (20.3%), getting free male circumcision medical services (16.8%), penile hygiene (9.8%), cosmetic reasons (7.7%), and peer influence (2.8%). The reasons of not accepting MC included no redundant foreskin problem (55.3%), inconvenient (12.1%), useless (8.8%), surgical complication (7.4%), and doubts about the effectiveness of MC as a HIV/STI prevention method (7.0%).

## 4. Discussion

This study revealed an alarming prevalence of HIV among MSM in Chengdu, substantially higher than that among other vulnerable groups, for example, injection drug users (3.9%) and female sex workers (0.8%) in Chengdu [27] and higher than the overall prevalence among MSM in a large national survey across 61 cities [6]. The HIV prevalence in this study is similar to that reported from South and Southeast Asia (range: 14% to 18%) [28]. In the last decade, HIV prevalence among MSM in Chengdu has progressively increased, which is consistent with the national trend of homosexual transmission [29]. A significant epidemiological change is that unprotected male-to-male sex replaced IDU as the predominant mode of transmission for HIV in this large metropolitan city. Our finding underscores the urgent need to strategically target intervention prevention efforts toward MSMs.

STIs have been associated with biological risk for HIV infection in MSM, notably syphilis and infection with herpes simplex virus type 2, and more recently anal infection with human papillomavirus [30]. High rates of undiagnosed and untreated syphilis are associated with the substantially higher rates of HIV infection [31]. In our study, syphilis prevalence is 15.9%, which was higher than that of the national survey [6]. After controlling for other risk factors, participants who were infected with syphilis were six times more likely to be infected by HIV. The finding of this study highlighted the needs for the treatment of STIs as one of the components of a comprehensive HIV prevention strategy.

The disproportionate HIV disease burden in MSM is explained largely by the high per-act and per-partner transmission probability of HIV transmission in receptive anal sex [28]. This study also shows that multiple male sex partners and receptive anal intercourse are associated with HIV infection. The participants in this study had an average of two male partners in the preceding 6 months. Of great concern, more than 5% of respondents reported having had at least ten male partners in the same time period. Besides, about two-thirds of participants engaged in IAI and 65.4% did not use condoms consistently. The finding of common unprotected receptive anal intercourse and multiple sex partners portend the high risk of continuous rapid expansion of the HIV epidemic among the MSM community in Chengdu.

In our study, the coverage of a single intervention was about 28% to 58%, which were much less than 60–80% needed to have an effect on the HIV epidemic [32]. Multiple logistic regression result showed that those who received the peer education in the last year had lower chances to infect HIV. The significant relationship between peer education and HIV intervention may be contributed to the relative high coverage of peer education service. This finding underscored the need for scaling up the intervention effort.

The study was the first in Sichuan to assess the frequency of male circumcision and its association with HIV infection among MSM. One-fifth of participants reported having been circumcised, which was similar to the finding in Beijing, China [33] and much lower than the male circumcision prevalence (79%) in US [34]. When comparing self-reported and genital examination, the study found that participant can accurately report their circumcision status, which was consistent with an Australia's study [35]. Over half uncircumcised respondents in our study indicated that they would be willing to be circumcised, which was similar to the reports in Beijing, China [33] and other countries [36]. The relative low prevalence of male circumcision and a high willingness to be circumcised could be the evidence of the feasibility to promote MC as an intervention component of the multifaceted intervention strategy among MSM in Chengdu.

This study did not demonstrate an independent association of male circumcision with HIV infection whether we restricted the sample to men who engage in RAI or IAI. This finding is consistent with the findings from a clinical trial among MSM [37]. In contrast, in this study, being circumcised was associated with a lower frequency of syphilis among MSM (especially among those who engaged in IAI), which is consistent with a prospective study to assess circumcision status and a broad range of STIs among MSM [21]. Circumcision is unlikely to reduce URAI risk but could partially protect against HIV for MSM practicing unprotected insertive anal intercourse (UIAI) [22]. Consistent with previous research, our study found an association between male circumcision and lower frequency of syphilis among MSM (especially among those who engaged in IAI). Further studies with more incident HIV infections and among men who are exclusively insertive partners will likely provide greater power to determine if circumcision is associated with lower rates of HIV infection among MSM who engage in insertive anal sex with HIV-infected partners among MSM in Chengdu.

This study has limitations. The study applied the snowball sampling method. Participants in the study may not be representative because of the biases associated with snowball sampling. However, compared with our previous study [10], the present study started from more seeds (eighteen) and had more diversity in demographic characteristics, especially with regard to age and education. With the exception of the clinical examination and HIV, syphilis serological testing, this study relied on self-reported data. Risk behaviors could be over- or underestimated due to social desirability regarding reporting. Additionally, the nature of the cross sectional design precluded the ascertainment of causality. This study did not find an independent association of circumcision with HIV infection, which may be caused by our use of prevalent cases.

In summary, this study demonstrated an alarming prevalence of HIV and syphilis among MSM in Chengdu. The common practice of unprotected receptive anal intercourse and multiple male sexual partnerships in our study population highlight the urgency for an effective comprehensive HIV prevention strategy, including the promotion of safer sex practices, treatment for STIs, the promotion of correct and consistent condom use, and the provision of HIV testing and antiretroviral therapy. Further research will shed more light on optimal ways and desirability of integrating male circumcision into a comprehensive prevention package to restrain the rapid expansion of the HIV epidemic among MSM in Chengdu, China.

## Figures and Tables

**Table 1:** Demographics, HIV/syphilis Infection, and risk factors among men who have sex with men in Chengdu, China, 2009.

Variables	*N* (%)	HIV (*N*, %)	Prevalence (%)
*Demographics *			
Age (years)			
18–24	226 (39.7)	29 (38.2)	12.8
25–34	210 (36.8)	26 (34.2)	12.4
≥35	134 (23.5)	21 (27.6)	15.7
Refused/missing	0	0	
Education			
Junior high	102 (17.9)	24 (31.6)	23.5
Senior high	130 (22.8)	22 (28.9)	16.9
College or higher	338 (59.3)	30 (39.5)	8.9
Refused/missing	0	0	
Marital status			
Never married	407 (71.4)	49 (64.5)	12.0
Married	76 (13.3)	10 (13.2)	13.2
Live with a partner (male/female)	44 (7.7)	8 (10.5)	5.9
Divorced/widowed	43 (7.5)	9 (11.8)	6.3
Refused/missing	0	0	
Household registration			
Local resident	261 (45.8)	26 (34.2)	10.0
Nonlocal resident	309 (54.2)	50 (65.8)	16.2
Refused/missing	0	0	
Time period of living in Chengdu			
0-1 years	61 (10.7)	9 (11.8)	14.8
1-2 years	54 (9.5)	8 (10.5)	14.8
≥2 years	455 (79.8)	59 (77.6)	13.0
Refused/missing	0	0	
Self-identified sexual orientation			
Homosexual	372 (65.3)	49 (64.5)	13.2
Heterosexual	7 (1.2)	0 (0)	0
Bisexual	191 (33.5)	27 (35.5)	14.1
Refused/missing	0	0	
*Sexual behavior in the past 6 months *			
Venues for finding sex partners			
Bars/tea houses/dance halls	104 (18.2)	16 (21.1)	15.4
Public bathhouses/saunas	60 (10.5)	11 (14.5)	5.0
Parks/public restroom	31 (5.4)	6 (7.9)	7.2
Internet sites	337 (59.1)	41 (53.9)	12.2
Refused/missing	38 (6.7)	2 (2.6)	
Anal sex with men			
Exclusively insertive	178 (31.2)	13 (17.1)	7.3
Predominantly insertive	132 (23.2)	19 (25.0)	14.4
Exclusively receptive	102 (17.9)	20 (25.3)	19.6
Predominantly receptive	108 (18.9)	22 (28.9)	20.4
Refused/missing	50 (8.8)	2 (2.6)	
Buy male sex			
No	549 (96.3)	72 (94.7)	13.1
Yes	21 (3.7)	4 (5.3)	19.0
Refused/missing	0	0	
Sell sex to a male			
No	545 (95.6)	70 (92.1)	12.8
Yes	25 (4.4)	6 (7.9)	24.0
Refused/missing			
Number of male sex partners			
0	49 (8.6)	2 (2.6)	4.1
1	178 (31.2)	15 (19.7)	8.4
2–9	304 (53.3)	47 (61.8)	15.5
≥10	32 (5.6)	11 (14.5)	34.4
Refused/missing	7 (1.2)	2 (2.6)	
Consistent condom use with male partner			
Always	321 (51.1)	55 (72.3)	17.1
Sometimes or never	200 (34.7)	19 (25.0)	9.5
Refused/missing	49 (14.2)	2 (2.6)	
Number of female sex partners			
0	476 (83.5)	67 (88.2)	14.1
1	67 (11.8)	5 (6.6)	7.5
≥2	27 (4.7)	4 (5.3)	14.8
Refused/missing	0	0	
*HIV knowledge *			
Correctly answered questions about HIV			
No	261 (45.8)	50 (65.8)	19.1
Yes	309 (54.2)	26 (34.2)	8.4
Refused/missing	0	0	
*Biological outcome *			
Syphilis			
Negative	477 (83.7)	42 (55.3)	8.8
Positive	90 (15.8)	34 (44.7)	37.8
Refused/missing	3 (5.3%)	0	

**Table 2:** HIV/syphilis infection, HIV prevention services and male circumcision practices and preferences among men who have sex with men in Chengdu, China, 2009.

Variables	*N* (%)	HIV (*N*, %)	Prevalence (%)
*Received HIV prevention services in the past year *			
Free condom			
No	282 (49.5)	39 (51.3)	13.8
Yes	288 (50.5)	37 (48.7)	12.8
Refused/missing	0	0	
Free lubricants			
No	307 (53.4)	39 (51.3)	12.7
Yes	263 (46.1)	37 (48.7)	14.1
Refused/missing	0	0	
Peer education			
No	239 (41.9)	43 (56.6)	18.0
Yes	331 (58.1)	33 (43.4)	10.0
Refused/missing	0	0	
STD test or treatment			
No	412 (72.3)	62 (81.6)	15.0
Yes	158 (27.7)	14 (18.4)	8.8
Refused/missing	0	0	
HIV counseling or testing			
No	303 (53.2)	51 (67.1)	16.8
Yes	267 (46.8)	25 (32.9)	9.4
Refused/missing	0	0	
HIV/AIDS knowledge booklet			
No	248 (43.5)	45 (59.2)	18.1
Yes	322 (56.5)	31 (40.8)	9.6
Refused/missing	0	0	
*Male circumcision *			
Been circumcised			
No	504 (88.4)	67 (88.2)	13.3
Yes	66 (11.6)	9 (11.8)	13.6
Refused/missing	0	0	
Genital examination results			
Be circumcised	66 (11.6)	9 (11.8)	13.6
Phimosis	6 (1.1)	2 (2.6)	33.3
Redundant foreskin	164 (28.8)	19 (25.0)	11.6
Paraphimosis	66 (11.6)	7 (9.2)	10.6
Normal	268 (47.0)	39 (51.3)	14.6
Refused/missing	0	0	

**Table 3:** Risk factors associated with HIV infection among MSM in Chengdu, China, 2009.

Factors	Univariate		Multivariate	
	OR (95%CI)	*P*-value	AOR (95%CI)	*P* value
Highest education (versus Junior high)				
Senior high	0.7 (0.3–1.3)	0.2132	0.9 (0.4–1.9)	0.7197
College or higher	0.3 (0.2–0.6)	0.0001	0.4 (0.2–0.9)	0.0295
MSM anal sex (versus exclusively insertive)				
Predominantly insertive	2.1 (1.0–4.5)	0.0461	1.5 (0.6–3.5)	0.3663
Exclusively receptive	3.1 (1.5–6.6)	0.003	2.9 (1.2–6.9)	0.0196
Predominantly receptive	3.2 (1.6–6.8)	0.0017	3.9 (1.6–9.3)	0.0023
Number of male sex partners in the past 6 months (versus 1)				
2–9	4.3 (1.0–18.3)	0.0486	1.7 (0.8–3.5)	0.1328
≥10	12.3 (2.5–60.5)	0.002	6.2 (2.0–19.1)	0.0016
Peer education (yes versus no)	0.5 (0.3–0.8)	0.006	0.5 (0.3–0.8)	0.028
Syphilis (positive versus negative)	6.3 (3.7–10.7)	<0.0001	6.9 (3.5–13.5)	<0.0001

Note: Multivariable logistic regression analysis was applied; OR: odds ratio; 95%CI: confidence interval; AOR: adjusted odds ratio.

**Table 4:** Association between HIV, syphilis, and circumcision among MSM, Chengdu, China, 2009.

Prevalence	Total MSM (*n* = 520)			MSM engaged in EIAI or PIAI (*N* = 310)			MSM engaged in ERAI or PRAI (*N* = 210)		
	Circumcised (%) (*n* = 60)	Uncircumcised (*n* = 460)	*P*	Circumcised (*n* = 38)	Uncircumcised (*n* = 272)	*P*	Circumcised (*n* = 22)	Uncircumcised (*n* = 188)	*P*
HIV	13.6	13.3	0.939	15.8	9.6	0.237	13.6	20.7	0.434
Syphilis	7.6	17.5	0.05	5.3	16.7	0.046	13.6	17.6	0.656

Note: Chi Square test was applied; EIAI: exclusively insertive anal intercourse; PIAI: predominantly insertive anal intercourse; ERAI: exclusively receptive anal intercourse; PRAI: predominantly receptive anal intercourse; due to missing data, sample size may differ.

## References

[1] Centers for Disease Control (CDC) (1981). Follow-up on Kaposi's sarcoma and Pneumocystis pneumonia. *MMWR Morbidity and Mortality Weekly Report*.

[2] Beyrer C, Baral SD, Walker D, Wirtz AL, Johns B, Sifakis F (2010). The expanding epidemics of HIV type 1 among men who have sex with men in low-and middle-income countries: Diversity and consistency. *Epidemiologic Reviews*.

[3] Baral S, Sifakis F, Cleghorn F, Beyrer C (2007). Elevated risk for HIV infection among men who have sex with men in low- and middle-income countries 2000–2006: a systematic review. *PLoS Medicine*.

[4] Li H-M, Peng R-R, Li J (2011). HIV incidence among men who have sex with men in China: a meta-analysis of published studies. *PLoS ONE*.

[5] Gao L, Zhang L, Jin Q (2009). Meta-analysis: prevalence of HIV infection and syphilis among MSM in China. *Sexually Transmitted Infections*.

[6] Wu Z, Xu J, Liu E, Mao Y, Xiao Y, Sun X (2013). HIV and syphilis prevalence among men who have sex with men: a cross-sectional survey of 61 cities in China. *Clinical Infectious Diseases*.

[7] Zhang L, Chow EPF, Jing J, Zhuang X, Li X, He M (2013). HIV prevalence in China: integration of surveillance data and a systematic review. *The Lancet Infectious Diseases*.

[8] Chengdu Gay Community Care Organization (2007). *Study of MSM Population Size in Chengdu*.

[9] He Q, Wu X, Han D (2007). Analysis HIV infection and related risk behaviors among MSMS in Chengdu from 2004 to 2007. *Journal of Occupational Health and Damage*.

[10] Feng Y, Wu Z, Detels R (2010). HIV/STD prevalence among men who have sex with men in Chengdu, China and associated risk factors for HIV infection. *Journal of Acquired Immune Deficiency Syndromes*.

[11] Sullivan PS, Carballo-Dieguez A, Coates T, Goodreau SM, McGowan I, Sanders EJ (2012). Successes and challenges of HIV prevention in men who have sex with men. *The Lancet*.

[12] Hayes R, Watson-Jones D, Celum C, Van De Wijgert J, Wasserheit J (2010). Treatment of sexually transmitted infections for HIV prevention: end of the road or new beginning?. *AIDS*.

[13] Cohen MS, Chen YQ, McCauley M (2011). Prevention of HIV-1 infection with early antiretroviral therapy. *The New England Journal of Medicine*.

[14] Vermund SH, Hayes RJ (2013). Combination prevention: new hope for stopping the epidemic. *Current HIV/AIDS Reports*.

[15] Vermund SH (2013). Treatment as prevention for HIV in China. *The Lancet*.

[16] Bailey RC, Moses S, Parker CB (2007). Male circumcision for HIV prevention in young men in Kisumu, Kenya: a randomised controlled trial. *The Lancet*.

[17] Gray RH, Kigozi G, Serwadda D (2007). Male circumcision for HIV prevention in men in Rakai, Uganda: a randomised trial. *The Lancet*.

[18] Auvert B, Taljaard D, Lagarde E, Sobngwi-Tambekou J, Sitta R, Puren A (2005). Randomized, controlled intervention trial of male circumcision for reduction of HIV infection risk: the ANRS 1265 trial. *PLoS Medicine*.

[19] Sánchez J, Sal Y Rosas VG, Hughes JP (2011). Male circumcision and risk of HIV acquisition among MSM. *AIDS*.

[20] Lane T, Raymond HF, Dladla S (2011). High HIV prevalence among men who have sex with men in Soweto, South Africa: results from the Soweto men’s study. *AIDS and Behavior*.

[21] Templeton DJ, Jin F, Prestage GP (2009). Circumcision and risk of sexually transmissible infections in a community-based cohort of HIV negative homosexual men in Sydney, Australia. *Journal of Infectious Diseases*.

[22] Templeton DJ, Millett GA, Grulich AE (2010). Male circumcision to reduce the risk of HIV and sexually transmitted infections among men who have sex with men. *Current Opinion in Infectious Diseases*.

[23] Okwuosa TM (2009). Male circumcision for prevention of HIV transmission. *The Lancet*.

[24] Vermund SH, Qian H-Z (2008). Circumcision and HIV prevention among men who have sex with men: no final word. *Journal of the American Medical Association*.

[25] Ben K-L, Xu J-C, Lu L (2009). Male circumcision is an effective “surgical vaccine” for HIV prevention and reproductive health. *National Journal of Andrology*.

[26] Magnani R, Sabin K, Saidel T, Heckathorn D (2005). Review of sampling hard-to-reach and hidden populations for HIV surveillance. *AIDS*.

[27] Sichuan Center of Disease Control and Prevention (2009). *HIV/AIDS Survrillance Report in Sichuan Province*.

[28] Beyrer C, Baral SD, van Griensven F, Goodreau SM, Chariyalertsak S, Wirtz AL (2012). Global epidemiology of HIV infection in men who have sex with men. *The Lancet*.

[29] Ministry of Health (2011). *Peoples's Republic of China. 2011 estimates For the HIV/AIDS Epidemic in China*.

[30] Chin-Hong PV, Husnik M, Cranston RD (2009). Anal human papillomavirus infection is associated with HIV acquisition in men who have sex with men. *AIDS*.

[31] Millett GA, Peterson JL, Wolitski RJ, Stall R (2006). Greater risk for HIV infection of black men who have sex with men: a critical literature review. *American Journal of Public Health*.

[32] Adam PCG, De Wit JBF, Toskin I (2009). Estimating levels of HIV testing, HIV prevention coverage, HIV knowledge, and condom use among men who have sex with men (MSM) in low-income and middle-income countries. *Journal of Acquired Immune Deficiency Syndromes*.

[33] Ruan Y, Qian H-Z, Li D (2009). Willingness to be circumcised for preventing HIV among chinese men who have sex with men. *AIDS Patient Care and STDs*.

[34] Xu F, Markowitz LE, Sternberg MR, Aral SO (2007). Prevalence of circumcision and herpes simplex virus type 2 infection in men in the United States: The National Health and Nutrition Examination Survey (NHANES), 1999–2004. *Sexually Transmitted Diseases*.

[35] Templeton DJ, Mao L, Prestage GP, Jin F, Kaldor JM, Grulich AE (2008). Self-report is a valid measure of circumcision status in homosexual men. *Sexually Transmitted Infections*.

[36] Begley EB, Jafa K, Voetsch AC, Heffelfinger JD, Borkowf CB, Sullivan PS (2008). Willingness of men who have sex with men (MSM) in the United States to be circumcised as adults to reduce the risk of HIV infection. *PLoS ONE*.

[37] Gust DA, Wiegand RE, Kretsinger K (2010). Circumcision status and HIV infection among MSM: reanalysis of a Phase III HIV vaccine clinical trial. *AIDS*.

